# 1-{5-[(*E*)-(4-Propyl­phen­yl)diazen­yl]-2-hy­droxy­phen­yl}ethanone

**DOI:** 10.1107/S1600536811004910

**Published:** 2011-02-16

**Authors:** Serap Yazıcı, Çiğdem Albayrak, Ismail Gümrükçüoğlu, Ismet Şenel, Orhan Büyükgüngör

**Affiliations:** a Department of Physics, Faculty of Arts and Sciences, Ondokuz Mayıs University, TR-55139 Kurupelit–Samsun, Turkey; b Sinop Faculty of Education, Sinop University, TR-57000 Sinop, Turkey; c Department of Chemistry, Ondokuz Mayıs University, TR-55139 Kurupelit–Samsun, Turkey

## Abstract

The mol­ecular geometry of the title compound, C_17_H_18_N_2_O_2_, displays an *E* configuration with respect to the azo group. The dihedral angle between the aromatic rings is 10.39 (4)°. In the mol­ecule, an intra­molecular O—H⋯O hydrogen bond generates an *S*(6) ring motif.

## Related literature

For general background to azo compounds, see: Russ & Tappe (1994 ▶); Tsuda *et al.* (2000 ▶). For bond-length data, see: Allen *et al.* (1987 ▶); Deveci *et al.* (2005 ▶); Karadayı *et al.*, (2006 ▶); El-Ghamry *et al.* (2008 ▶); Albayrak *et al.*, 2009 ▶; Yazıcı *et al.* (2010 ▶).
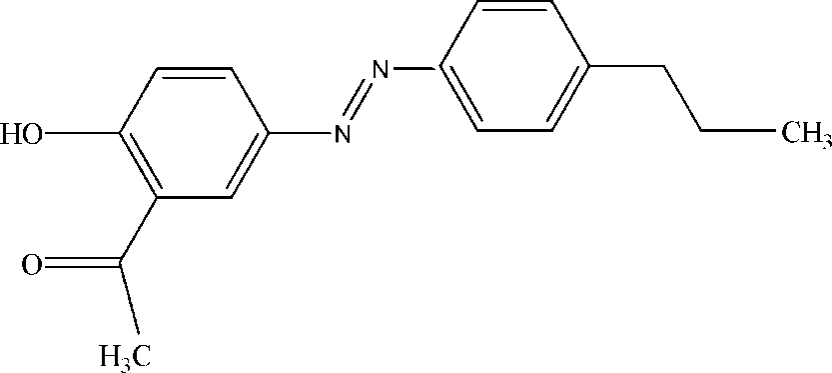


## Experimental

### 

#### Crystal data

C_17_H_18_N_2_O_2_
*M* *_r_* = 282.33
Monoclinic, 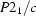

*a* = 14.8315 (5) Å
*b* = 7.5573 (2) Å
*c* = 13.5020 (4) Å
β = 102.578 (3)°
*V* = 1477.07 (8) Å^3^
*Z* = 4
Mo *K*α radiation
μ = 0.08 mm^−1^
*T* = 150 K
0.75 × 0.47 × 0.21 mm

#### Data collection

Stoe IPDS II diffractometer
Absorption correction: integration (*X-RED32*; Stoe & Cie, 2002 ▶) *T*_min_ = 0.946, *T*_max_ = 0.984
21625 measured reflections
3054 independent reflections
2680 reflections with *I* > 2σ(*I*)
*R*_int_ = 0.039

#### Refinement

*R*[*F* ^2^ > 2σ(*F* ^2^)] = 0.035
*wR*(*F* ^2^) = 0.097
*S* = 1.04
3054 reflections
195 parameters
H atoms treated by a mixture of independent and constrained refinement
Δρ_max_ = 0.21 e Å^−3^
Δρ_min_ = −0.16 e Å^−3^

### 

Data collection: *X-AREA* (Stoe & Cie, 2002 ▶); cell refinement: *X-AREA*; data reduction: *X-RED32* (Stoe & Cie, 2002 ▶); program(s) used to solve structure: *SHELXS97* (Sheldrick, 2008 ▶); program(s) used to refine structure: *SHELXL97* (Sheldrick, 2008 ▶); molecular graphics: *ORTEP-3 for Windows* (Farrugia, 1997 ▶); software used to prepare material for publication: *WinGX* (Farrugia, 1999 ▶).

## Supplementary Material

Crystal structure: contains datablocks I, global. DOI: 10.1107/S1600536811004910/bh2335sup1.cif

Structure factors: contains datablocks I. DOI: 10.1107/S1600536811004910/bh2335Isup2.hkl

Additional supplementary materials:  crystallographic information; 3D view; checkCIF report

## Figures and Tables

**Table 1:** Hydrogen-bond geometry (Å, °)

*D*—H⋯*A*	*D*—H	H⋯*A*	*D*⋯*A*	*D*—H⋯*A*
O1—H1⋯O2	0.921 (19)	1.675 (18)	2.5365 (13)	154.3 (16)

**Fig. 1.**
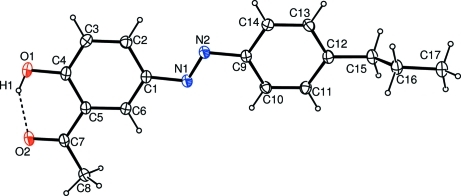
An ORTEP view of the title compound, with the atom-numbering scheme and 30% probability displacement ellipsoids.

## supplementary crystallographic information

### Comment

Azo colorants, which are characterized by one or more azo bonds, are the most versatile class of dyes. They are used in textiles, printing, cosmetics, drugs and other consumer goods (Russ & Tappe, 1994; Tsuda *et al.*, 2000).

A view of a molecule of the title compound, together with the atom-numbering scheme, is shown in Fig. 1. The title molecule adopts the *E* configuration with respect to N═N bridge and the C1—N1—N2—C9 torsion angle is -178.33 (8)°. The *A*/*B* and *B*/*C* dihedral angles between the *A* (C1···C6), *B* (C9···14) and *C* (C12/C15/C16/C17) fragments are 10.39 (4) and 76.04 (8)°, respectively.

The N1—C1 and N2—C9 bond lengths of 1.4203 (12) and 1.4271 (12) Å, respectively, indicate single-bond character, whereas the N1—N2 bond length of 1.2572 (12) Å indicates double-bond character. In the molecule, all bond lengths are in good agreement with those reported for other azo compounds (Allen *et al.*, 1987; Deveci *et al.*, 2005; El-Ghamry *et al.*, 2008; Albayrak *et al.*, 2009; Yazıcı *et al.*, 2010; Karadayı *et al.*, 2006). There is a strong intra-molecular hydrogen bond of 2.5365 (13) Å between atoms O1 and O2. The crystal packing is controlled by dipole-dipole and van der Waals interactions, and molecules are stacked along crystallographic [010] direction.

### Experimental

A mixture of 4-propylaniline (1.05 g, 7.8 mmol), water (20 ml) and concentrated hydrochloric acid (1.97 ml, 23.4 mmol) was stirred until a clear solution was obtained. This solution was cooled down to 0–5 °C and a solution of sodium nitrite (0.75 g 7.8 mmol) in water was added dropwise while the temperature was maintained below 5 °C. The resulting mixture was stirred for 30 min in an ice bath. 2-Hydroxyacetophenone (1.067 g, 7.8 mmol, solution at pH 9) was gradually added to a cooled solution of 4-propylbenzenediazonium chloride, prepared as described above, and the resulting mixture was stirred at 0–5 °C for 2 h in an ice bath. The product was recrystallized from ethanol to obtain solid (*E*)-2-acetyl-4-(4-propylphenyldiazenyl)phenol. Crystals were obtained after one day by slow evaporation from acetic acid (yield 45%, m.p. = 350–352 K).

### Refinement

All C-bonded H atoms were positioned with idealized geometry using a riding model, with C—H = 0.93–0.97 Å. Hydroxyl H atom H1 was found in a difference map and refined freely. All H atoms were refined with *U*_iso_=1.2*U*_eq_(parent atom) or *U*_iso_=1.5*U*_eq_(parent atom)

### Crystal data

**Table:**

C_17_H_18_N_2_O_2_	*F*(000) = 600
*M**_r_* = 282.33	*D*_x_ = 1.270 Mg m^−^^3^
Monoclinic, *P*2_1_/*c*	Melting point: 350 K
Hall symbol: -P 2ybc	Mo *K*α radiation, λ = 0.71073 Å
*a* = 14.8315 (5) Å	Cell parameters from 29224 reflections
*b* = 7.5573 (2) Å	θ = 1.5–28.0°
*c* = 13.5020 (4) Å	µ = 0.08 mm^−^^1^
β = 102.578 (3)°	*T* = 150 K
*V* = 1477.07 (8) Å^3^	Prism, brown
*Z* = 4	0.75 × 0.47 × 0.21 mm

### Data collection

**Table:**

Stoe IPDS II diffractometer	3054 independent reflections
Radiation source: fine-focus sealed tube	2680 reflections with *I* > 2σ(*I*)
graphite	*R*_int_ = 0.039
Detector resolution: 6.67 pixels mm^-1^	θ_max_ = 26.5°, θ_min_ = 2.8°
ω scans	*h* = −18→18
Absorption correction: integration (*X-RED32*; Stoe & Cie, 2002)	*k* = −9→9
*T*_min_ = 0.946, *T*_max_ = 0.984	*l* = −16→16
21625 measured reflections	

### Refinement

**Table:**

Refinement on *F*^2^	Primary atom site location: structure-invariant direct methods
Least-squares matrix: full	Secondary atom site location: difference Fourier map
*R*[*F*^2^ > 2σ(*F*^2^)] = 0.035	Hydrogen site location: inferred from neighbouring sites
*wR*(*F*^2^) = 0.097	H atoms treated by a mixture of independent and constrained refinement
*S* = 1.04	*w* = 1/[σ^2^(*F*_o_^2^) + (0.0487*P*)^2^ + 0.2885*P*] where *P* = (*F*_o_^2^ + 2*F*_c_^2^)/3
3054 reflections	(Δ/σ)_max_ < 0.001
195 parameters	Δρ_max_ = 0.21 e Å^−^^3^
0 restraints	Δρ_min_ = −0.16 e Å^−^^3^
0 constraints	

### Fractional atomic coordinates and isotropic or equivalent isotropic displacement parameters (Å^2^)

**Table:**

	*x*	*y*	*z*	*U*_iso_*/*U*_eq_	
C1	0.47997 (7)	0.68823 (13)	0.36509 (7)	0.0259 (2)	
C2	0.50206 (7)	0.61432 (14)	0.27782 (8)	0.0288 (2)	
H2	0.5600	0.5644	0.2815	0.035*	
C3	0.43876 (7)	0.61552 (14)	0.18743 (8)	0.0310 (2)	
H3	0.4540	0.5670	0.1299	0.037*	
C4	0.35131 (7)	0.68914 (14)	0.18103 (8)	0.0293 (2)	
C5	0.32685 (7)	0.76156 (13)	0.26807 (7)	0.0264 (2)	
C6	0.39293 (7)	0.75846 (13)	0.35970 (7)	0.0260 (2)	
H6	0.3780	0.8045	0.4180	0.031*	
C7	0.23300 (7)	0.83175 (13)	0.26066 (8)	0.0287 (2)	
C8	0.20412 (7)	0.89985 (15)	0.35252 (8)	0.0319 (2)	
H8A	0.1425	0.9460	0.3335	0.048*	
H8B	0.2455	0.9921	0.3829	0.048*	
H8C	0.2058	0.8053	0.4004	0.048*	
C9	0.68461 (7)	0.65001 (13)	0.55710 (7)	0.0253 (2)	
C10	0.65886 (7)	0.69262 (14)	0.64747 (8)	0.0281 (2)	
H10	0.5984	0.7262	0.6467	0.034*	
C11	0.72316 (7)	0.68489 (14)	0.73810 (8)	0.0290 (2)	
H11	0.7055	0.7149	0.7980	0.035*	
C12	0.81431 (7)	0.63289 (13)	0.74182 (8)	0.0277 (2)	
C13	0.83931 (7)	0.59345 (15)	0.65066 (8)	0.0310 (2)	
H13	0.8999	0.5611	0.6513	0.037*	
C14	0.77553 (7)	0.60153 (14)	0.55917 (8)	0.0298 (2)	
H14	0.7934	0.5746	0.4991	0.036*	
C15	0.88314 (7)	0.61869 (15)	0.84173 (8)	0.0323 (2)	
H15A	0.9176	0.5094	0.8424	0.039*	
H15B	0.8498	0.6120	0.8959	0.039*	
C16	0.95106 (7)	0.77208 (15)	0.86325 (8)	0.0332 (2)	
H16A	0.9171	0.8825	0.8588	0.040*	
H16B	0.9881	0.7742	0.8122	0.040*	
C17	1.01453 (8)	0.75689 (16)	0.96802 (8)	0.0364 (3)	
H17A	1.0562	0.8557	0.9791	0.055*	
H17B	1.0492	0.6489	0.9722	0.055*	
H17C	0.9782	0.7565	1.0188	0.055*	
N1	0.54195 (6)	0.69552 (12)	0.46137 (6)	0.0276 (2)	
N2	0.62283 (6)	0.64756 (12)	0.45996 (6)	0.0276 (2)	
O1	0.29175 (6)	0.68415 (12)	0.09043 (6)	0.0392 (2)	
O2	0.17695 (6)	0.83191 (12)	0.17831 (6)	0.0416 (2)	
H1	0.2391 (13)	0.734 (2)	0.1040 (13)	0.074 (5)*	

### Atomic displacement parameters (Å^2^)

**Table:**

	*U*^11^	*U*^22^	*U*^33^	*U*^12^	*U*^13^	*U*^23^
C1	0.0254 (5)	0.0280 (5)	0.0231 (5)	−0.0024 (4)	0.0022 (4)	0.0021 (4)
C2	0.0267 (5)	0.0311 (5)	0.0286 (5)	−0.0007 (4)	0.0062 (4)	0.0006 (4)
C3	0.0357 (6)	0.0334 (5)	0.0244 (5)	−0.0029 (4)	0.0076 (4)	−0.0017 (4)
C4	0.0333 (5)	0.0295 (5)	0.0219 (5)	−0.0040 (4)	−0.0010 (4)	0.0019 (4)
C5	0.0271 (5)	0.0260 (5)	0.0240 (5)	−0.0016 (4)	0.0012 (4)	0.0025 (4)
C6	0.0265 (5)	0.0276 (5)	0.0227 (5)	−0.0019 (4)	0.0028 (4)	0.0002 (4)
C7	0.0279 (5)	0.0260 (5)	0.0283 (5)	−0.0010 (4)	−0.0024 (4)	0.0037 (4)
C8	0.0261 (5)	0.0347 (6)	0.0330 (6)	0.0025 (4)	0.0023 (4)	0.0038 (4)
C9	0.0239 (5)	0.0255 (5)	0.0250 (5)	−0.0010 (4)	0.0019 (4)	0.0016 (4)
C10	0.0228 (5)	0.0324 (5)	0.0291 (5)	0.0007 (4)	0.0053 (4)	0.0017 (4)
C11	0.0282 (5)	0.0331 (5)	0.0253 (5)	−0.0022 (4)	0.0050 (4)	0.0013 (4)
C12	0.0266 (5)	0.0254 (5)	0.0285 (5)	−0.0028 (4)	0.0003 (4)	0.0037 (4)
C13	0.0226 (5)	0.0343 (5)	0.0344 (6)	0.0035 (4)	0.0029 (4)	0.0005 (4)
C14	0.0268 (5)	0.0343 (5)	0.0281 (5)	0.0024 (4)	0.0057 (4)	−0.0016 (4)
C15	0.0298 (5)	0.0336 (6)	0.0297 (5)	−0.0012 (4)	−0.0019 (4)	0.0058 (4)
C16	0.0316 (5)	0.0325 (5)	0.0311 (5)	−0.0010 (4)	−0.0032 (4)	0.0037 (4)
C17	0.0321 (6)	0.0419 (6)	0.0311 (6)	−0.0022 (5)	−0.0019 (4)	0.0017 (5)
N1	0.0236 (4)	0.0315 (4)	0.0259 (4)	0.0001 (3)	0.0015 (3)	0.0012 (3)
N2	0.0238 (4)	0.0314 (4)	0.0265 (4)	0.0001 (3)	0.0026 (3)	0.0014 (3)
O1	0.0409 (5)	0.0493 (5)	0.0220 (4)	0.0021 (4)	−0.0050 (3)	−0.0028 (3)
O2	0.0359 (4)	0.0484 (5)	0.0326 (4)	0.0095 (4)	−0.0100 (3)	−0.0021 (4)

### Geometric parameters (Å, °)

**Table:**

C1—C6	1.3830 (14)	C10—C11	1.3792 (14)
C1—C2	1.4057 (14)	C10—H10	0.9300
C1—N1	1.4203 (12)	C11—C12	1.3982 (14)
C2—C3	1.3682 (14)	C11—H11	0.9300
C2—H2	0.9300	C12—C13	1.3930 (15)
C3—C4	1.3965 (15)	C12—C15	1.5079 (13)
C3—H3	0.9300	C13—C14	1.3840 (14)
C4—O1	1.3444 (12)	C13—H13	0.9300
C4—C5	1.4135 (15)	C14—H14	0.9300
C5—C6	1.4013 (13)	C15—C16	1.5217 (15)
C5—C7	1.4725 (14)	C15—H15A	0.9700
C6—H6	0.9300	C15—H15B	0.9700
C7—O2	1.2349 (12)	C16—C17	1.5233 (14)
C7—C8	1.4893 (15)	C16—H16A	0.9700
C8—H8A	0.9600	C16—H16B	0.9700
C8—H8B	0.9600	C17—H17A	0.9600
C8—H8C	0.9600	C17—H17B	0.9600
C9—C14	1.3917 (14)	C17—H17C	0.9600
C9—C10	1.3933 (14)	N1—N2	1.2572 (12)
C9—N2	1.4271 (12)	O1—H1	0.919 (19)
			
C6—C1—C2	119.58 (9)	C10—C11—C12	121.34 (10)
C6—C1—N1	116.35 (9)	C10—C11—H11	119.3
C2—C1—N1	124.06 (9)	C12—C11—H11	119.3
C3—C2—C1	120.34 (10)	C13—C12—C11	118.02 (9)
C3—C2—H2	119.8	C13—C12—C15	121.11 (9)
C1—C2—H2	119.8	C11—C12—C15	120.87 (9)
C2—C3—C4	120.37 (10)	C14—C13—C12	121.16 (9)
C2—C3—H3	119.8	C14—C13—H13	119.4
C4—C3—H3	119.8	C12—C13—H13	119.4
O1—C4—C3	117.55 (9)	C13—C14—C9	120.04 (10)
O1—C4—C5	122.04 (10)	C13—C14—H14	120.0
C3—C4—C5	120.39 (9)	C9—C14—H14	120.0
C6—C5—C4	118.09 (9)	C12—C15—C16	114.06 (8)
C6—C5—C7	122.34 (9)	C12—C15—H15A	108.7
C4—C5—C7	119.54 (9)	C16—C15—H15A	108.7
C1—C6—C5	121.21 (9)	C12—C15—H15B	108.7
C1—C6—H6	119.4	C16—C15—H15B	108.7
C5—C6—H6	119.4	H15A—C15—H15B	107.6
O2—C7—C5	120.17 (10)	C15—C16—C17	111.68 (9)
O2—C7—C8	119.36 (9)	C15—C16—H16A	109.3
C5—C7—C8	120.46 (9)	C17—C16—H16A	109.3
C7—C8—H8A	109.5	C15—C16—H16B	109.3
C7—C8—H8B	109.5	C17—C16—H16B	109.3
H8A—C8—H8B	109.5	H16A—C16—H16B	107.9
C7—C8—H8C	109.5	C16—C17—H17A	109.5
H8A—C8—H8C	109.5	C16—C17—H17B	109.5
H8B—C8—H8C	109.5	H17A—C17—H17B	109.5
C14—C9—C10	119.50 (9)	C16—C17—H17C	109.5
C14—C9—N2	116.13 (9)	H17A—C17—H17C	109.5
C10—C9—N2	124.35 (9)	H17B—C17—H17C	109.5
C11—C10—C9	119.92 (9)	N2—N1—C1	113.86 (8)
C11—C10—H10	120.0	N1—N2—C9	113.96 (8)
C9—C10—H10	120.0	C4—O1—H1	103.0 (11)
			
C6—C1—C2—C3	1.58 (15)	N2—C9—C10—C11	177.63 (9)
N1—C1—C2—C3	−178.81 (9)	C9—C10—C11—C12	−0.73 (15)
C1—C2—C3—C4	−0.41 (15)	C10—C11—C12—C13	1.73 (15)
C2—C3—C4—O1	−179.12 (9)	C10—C11—C12—C15	−177.81 (9)
C2—C3—C4—C5	−0.68 (16)	C11—C12—C13—C14	−1.41 (15)
O1—C4—C5—C6	178.96 (9)	C15—C12—C13—C14	178.13 (9)
C3—C4—C5—C6	0.59 (15)	C12—C13—C14—C9	0.11 (16)
O1—C4—C5—C7	0.93 (15)	C10—C9—C14—C13	0.93 (15)
C3—C4—C5—C7	−177.43 (9)	N2—C9—C14—C13	−177.46 (9)
C2—C1—C6—C5	−1.67 (15)	C13—C12—C15—C16	77.33 (13)
N1—C1—C6—C5	178.69 (9)	C11—C12—C15—C16	−103.14 (12)
C4—C5—C6—C1	0.59 (15)	C12—C15—C16—C17	176.16 (9)
C7—C5—C6—C1	178.56 (9)	C6—C1—N1—N2	−172.82 (9)
C6—C5—C7—O2	180.00 (10)	C2—C1—N1—N2	7.55 (14)
C4—C5—C7—O2	−2.06 (15)	C1—N1—N2—C9	−178.33 (8)
C6—C5—C7—C8	−1.21 (15)	C14—C9—N2—N1	−178.69 (9)
C4—C5—C7—C8	176.72 (9)	C10—C9—N2—N1	3.01 (14)
C14—C9—C10—C11	−0.62 (15)		

### Hydrogen-bond geometry (Å, °)

**Table:**

*D*—H···*A*	*D*—H	H···*A*	*D*···*A*	*D*—H···*A*
O1—H1···O2	0.921 (19)	1.675 (18)	2.5365 (13)	154.3 (16)
